# Performance of machine learning–based prediction models for hypoglycemia in Chinese patients with diabetes: A systematic review and meta-analysis

**DOI:** 10.1371/journal.pone.0358876

**Published:** 2026-09-22

**Authors:** Jinhua Yan, Yanping Song, Yangmei Du, Fanmin Li

**Affiliations:** Department of General Practice, the People’s Hospital of Leshan, Leshan, China; The Second Affiliated Hospital of Shandong First Medical University, CHINA

## Abstract

**Objectives:**

This study aimed to systematically evaluate the predictive performance of machine learning (ML)–based models for predicting hypoglycemia in Chinese patients with diabetes.

**Methods:**

We systematically searched PubMed, Embase, Web of Science, the Cochrane Library, CINAHL, CNKI, and Wanfang databases from inception to February 2026. Eligible studies focused on the development or validation of ML–based models for predicting hypoglycemia in Chinese patients with diabetes. Study selection and data extraction were performed independently by two reviewers. Information on study characteristics, modeling approaches, predictors, validation methods, and model performance was collected. The area under the receiver operating characteristic curve (AUC) was synthesized using a random-effects model. Study quality was assessed using the Prediction Model Risk of Bias Assessment Tool (PROBAST).

**Results:**

A total of 13 studies were included, and the pooled prevalence of hypoglycemia was 25% (95% CI: 17%–33%). The overall pooled area under the receiver operating characteristic curve (AUC) was 0.90 (95% CI: 0.87–0.93). Subgroup analyses by modeling algorithms showed pooled AUCs of 0.89 for extreme gradient boosting (XGBoost), 0.88 for random forest (RF), 0.85 for support vector machine (SVM), 0.84 for Light Gradient Boosting Machine (LightGBM), 0.83 for logistic regression (LR), and 0.81 for decision tree (DT) models. Common predictors included age, insulin use, body mass index, HbA1c, creatinine, and history of hypoglycemia.

**Conclusion:**

We attempted to provide a comprehensive overview of machine learning–based prediction models for hypoglycemia in patients with diabetes. Research in this field remains at an early stage, although several models with good discriminatory performance have been reported. Methodological limitations and insufficient validation were observed in many studies. Concerns regarding model robustness and interpretability also exist. More efforts to develop reliable and interpretable models and to promote their application in clinical practice for early risk identification are needed.

## 1 Introduction

Diabetes mellitus is one of the most prevalent chronic metabolic diseases worldwide [1]. According to the International Diabetes Federation (IDF) Diabetes Atlas, approximately 148 million adults in China are living with diabetes, representing the largest population of individuals with diabetes worldwide [2,3]. China has the largest population of individuals with diabetes worldwide, with clinical characteristics and diabetes management contexts that may differ from those reported in Western countries. Chinese patients with type 2 diabetes often develop diabetes at relatively lower BMI levels and exhibit distinct metabolic characteristics [4]. In addition, differences in dietary patterns, glucose monitoring accessibility, and glucose-lowering medication use [5] may contribute to variations in hypoglycemia risk profiles and predictive variable distributions.

Hypoglycemia is one of the most common and potentially underrecognized complications during glucose-lowering therapy [6]. Among patients receiving insulin or oral hypoglycemic agents, the incidence of mild-to-moderate hypoglycemia has been reported to reach 50% and 45%, respectively, while severe hypoglycemia occurs in approximately 21% and 6% of these patients [7]. Mild hypoglycemia can impair quality of life and treatment adherence, whereas severe hypoglycemia is associated with increased risks of falls, cardiovascular events, and mortality [8,9]. The reported case fatality rate of hypoglycemia is approximately 12.9%, rising to 24.9% in severe cases [10]. Early identification of individuals at high risk is therefore essential to prevent hypoglycemic events and improve diabetes management [11].

Risk prediction models have been developed to assist clinicians in identifying patients vulnerable to hypoglycemia. Conventional models generally incorporate demographic characteristics, clinical, and treatment-related variables [12]. However, their predictive performance and generalizability across populations remain limited [13]. As diabetes management often involves complex treatment adjustments and heterogeneous patient characteristics, more refined approaches to risk assessment are needed. With the increasing availability of complex clinical data, machine learning (ML)–based models have been applied to hypoglycemia prediction. By incorporating diverse clinical information, these models can capture complex patterns and improve risk stratification in diabetes care [14–16]. Previous systematic reviews, such as that conducted by Liu et al.[17], have summarized the overall evidence. However, the performance and clinical applicability of ML-based prediction models among Chinese patients with diabetes have not been evaluated. Therefore, this systematic review focuses on Chinese patients with diabetes to to evaluate the performance, methodological quality, and clinical applicability of ML-based prediction models for hypoglycemia.

## 2 Material

### 2.1 Protocol and registration

This meta-analysis was registered in PROSPERO (CRD420261338933) and conducted in accordance with the Preferred Reporting Items for Systematic Reviews and Meta-Analyses of Diagnostic Test Accuracy Studies (PRISMA-DTA) guidelines [18].

### 2.2 Literature search

A comprehensive search of both English and Chinese literature was conducted in the databases PubMed, Embase, Web of Science, the Cochrane Library, CINAHL, China National Knowledge Infrastructure (CNKI), and the Wanfang Database. In addition, the reference lists of all included studies were manually screened to identify any potentially relevant articles. The search strategy combined Medical Subject Headings (MeSH) terms and free-text keywords related to hypoglycemia, diabetes, and ML –based prediction models. Key search terms included “hypoglycemia”, “diabetes mellitus”, “machine learning”, “artificial intelligence”, “deep learning”, “prediction model”, “risk factors”, and “risk prediction”. Boolean operators (“OR” and “AND”) were used to combine search terms across different concept groups. The search covered studies from database inception to February 2026, and only full-text original research articles were considered. All retrieved records were imported into NoteExpress software for literature management and duplicate removal.

### 2.3 Eligibility criteria

We applied the Population, Index, Comparator, Outcome, Timing, and Setting (PICOTS) framework [19] to define the eligibility criteria for this review: P (Chinese patients with diabetes mellitus), I (Prediction models), C (not applicable), O (hypoglycemia), T (prediction during the course of diabetes), and S (clinical settings, including inpatient and outpatient care).

The inclusion criteria were as follows: [1] studies involving Chinese patients with type 1 or type 2 diabetes; [2] studies developing ML–based models for hypoglycemia prediction; [3] studies reporting the area under the receiver operating characteristic curve (AUC) as a measure of model performance; [4] observational study designs (e.g., cohort or case-control studies); and [5] articles published in English or Chinese. The exclusion criteria were: [1] studies assessing risk factors without developing prediction models; [2] studies with incomplete data or unavailable full texts; and [3] reviews, editorials, letters, case reports, conference abstracts, or other non-original studies.

### 2.4 Study selection

Two reviewers independently screened the titles and abstracts of all retrieved records to identify potentially eligible studies and remove duplicate entries. The full texts of the remaining articles were then assessed for eligibility according to the predefined inclusion and exclusion criteria. Reasons for exclusion were documented during the full-text screening stage. Any disagreements were resolved through discussion, and a third reviewer was consulted when consensus could not be reached.

### 2.5 Data extraction

Two authors (Yan and Song) independently extracted data from all eligible studies using a standardized data extraction form developed based on the CHARMS checklist [20](Checklist for Critical Appraisal and Data Extraction for Systematic Reviews of Prediction Modelling Studies). The extracted information included the first author, publication year, study design, study setting, dataset characteristics, modeling methods, validation approach, model performance, and predictors. All extracted data were cross-checked against the original articles. Any discrepancies were resolved through discussion, and if consensus could not be reached, a third reviewer was consulted for adjudication.

### 2.6 Quality appraisal

The risk of bias and applicability of the included studies were evaluated using the Prediction Model Risk of Bias Assessment Tool (PROBAST), a methodological instrument designed for studies developing or validating prediction models [21]. PROBAST assesses risk of bias across four domains: participants, predictors, outcome, and analysis. Each domain contains several signaling questions that are rated as “yes/probably yes,” “no/probably no,” or “no information,” and the responses are used to determine whether the domain has a low, high, or unclear risk of bias. A domain was judged to be at low risk only when all signaling questions were answered positively; otherwise, it was considered to have high or unclear risk. Applicability concerns were evaluated in three domains—participants, predictors, and outcome—based on criteria aligned with the risk-of-bias assessment [22]. Two reviewers independently conducted the quality assessment, and any disagreements were resolved through discussion or consultation with a third reviewer.

### 2.7 Data synthesis

In this study, STATA 18.0 software was used to conduct the meta-analysis. Heterogeneity among the included studies was assessed using the Higgins I² statistic. Given the inherent heterogeneity of prediction modeling studies, a random-effects meta-analysis was performed [23]. The AUC was used as the primary measure of predictive performance, and pooled estimates were reported with corresponding 95% confidence intervals(CI). An AUC between 0.7 and 0.9 was considered to indicate moderate predictive accuracy, while an AUC greater than 0.9 indicated high predictive accuracy. Subgroup analyses were performed based on relevant study characteristics when at least two studies were available. Sensitivity analysis was conducted by sequentially excluding individual studies to assess the robustness of the pooled estimates. Publication bias was evaluated using Egger’s test and visually assessed with funnel plots [24]. A two-sided P value < 0.05 was considered statistically significant.

## 3 Results

### 3.1 Study selection

**Fig 1:**
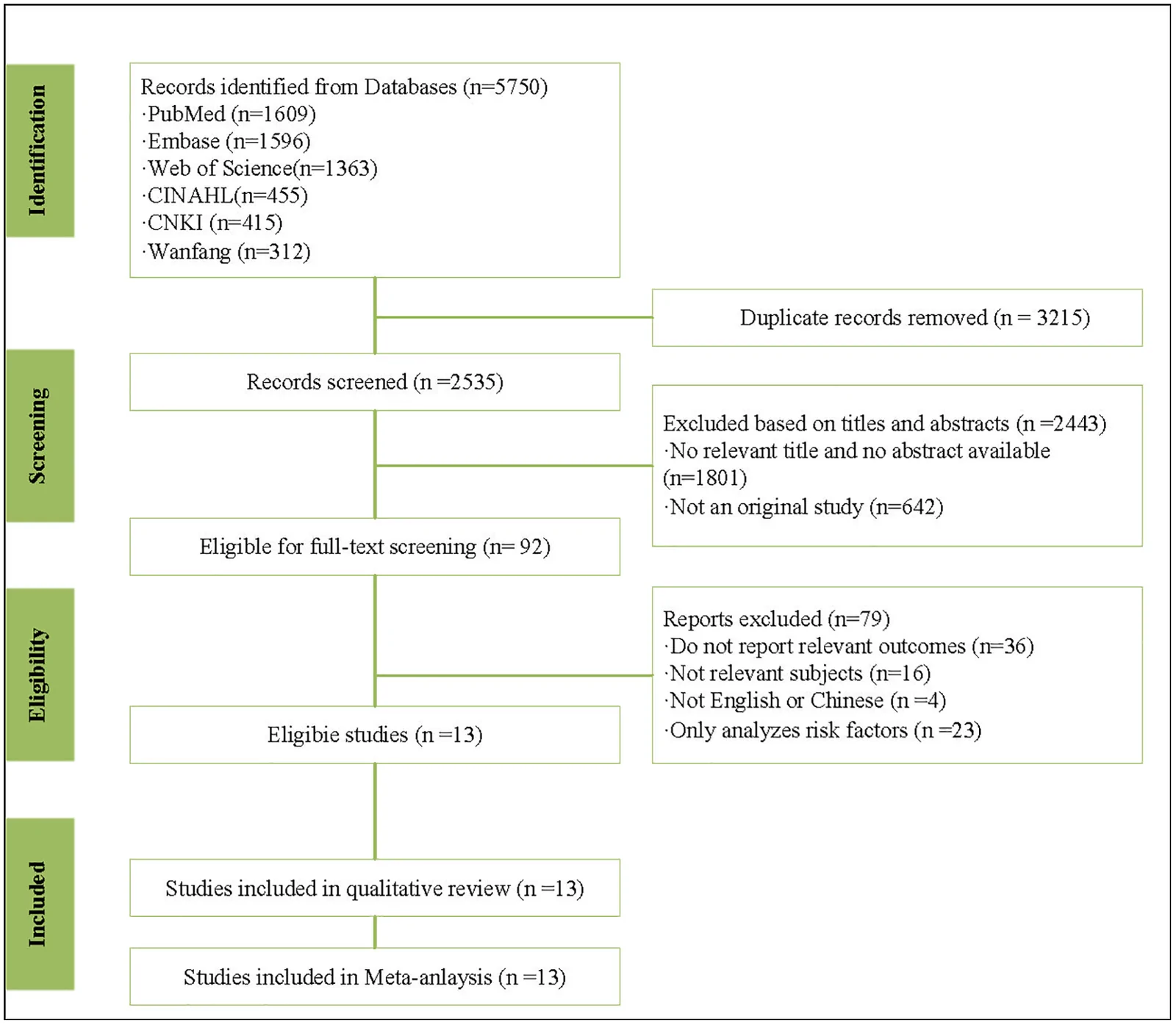
The article selection process.

A total of 5,450 records were identified through electronic database searches, including PubMed (n = 1,609), Embase (n = 1,596), Web of Science (n = 1,363), CINAHL (n = 455), CNKI (n = 415), and Wanfang (n = 312). After removing 3,215 duplicate records, 2,235 articles remained for title and abstract screening, of which 2,143 were excluded for not meeting the inclusion criteria. The full texts of 92 articles were subsequently assessed for eligibility. Among them, 79 studies were excluded for the following reasons: not reporting relevant outcomes (n = 36), irrelevant study populations (n = 16), publication in languages other than English or Chinese (n = 4), and analyses focusing only on risk factors (n = 23). Ultimately, 13 studies were included in the final meta-analysis. The study selection process is illustrated in the PRISMA flow diagram (Fig 1).

### 3.2 Characteristics of the included primary studies

A total of 13 studies investigating ML–based prediction models for hypoglycemia in patients with diabetes were included in this review. The studies were published between 2022 and 2026 and were all conducted in China. Most studies used a retrospective design (n = 11), while two studies adopted a prospective design. The prevalence of hypoglycemia reported in the included studies ranged from 4% to 46.93%. The majority of studies focused on patients with type 2 diabetes mellitus (T2DM), whereas 3 studies included both type 1 diabetes mellitus (T1DM) and T2DM populations. Prediction horizons varied across studies, ranging from 30 minutes to 12 months, with most models designed for short-term hypoglycemia prediction. Most studies were conducted in single-center settings (n = 11), while two studies used multicenter data. Detailed characteristics of the included studies are presented in Table 1.

**Table 1:** Basic characteristics of the included literature.

Study	Year	Study design	Prevalence	Type of Diabetes	Prediction horizon	Center type	Predictors
Zhang et al.[25]	2023	prospective study	42.67%	T2DM	1 week	Single-center	Number of hypoglycemic episodes, High-density lipoprotein cholesterol (HDL-C), heart disease, diabetes education, combination therapy, Age, duration of diabetes, Glycated hemoglobin (HbA1c), and Sex
Zheng et al.[26]	2023	retrospective study	18.79%	T2DM	1 week	Single-center	Insulin, Creatinine, Glycated serum protein (GSP), Blood urea nitrogen (BUN), C-peptide (CP), Total bilirubin (TBIL), and HDL-C
Niu et al.[27]	2023	retrospective study	46.93%	T2DM	1 week	Single-center	BMI, family history of diabetes, length of hospital stay(LOS), duration of diabetes, history of hypoglycemia, Insulin, Urinary albumin-to-creatinine ratio (UACR), Triglycerides (TG), minimum blood glucose (BGmin), Alanine aminotransferase/ Aspartate aminotransferase ratio (ALT/AST), and daily frequency of blood glucose monitoring
Zuo et al [28]	2023	retrospective study	–	T2DM	30min	Single-center	Age, Height, Weight, BMI, Meal omission, Systolic blood pressure (SBP), Creatinine (Cr), TG, HDL-C, Low-density lipoprotein cholesterol (LDL-C), Total cholesterol (TC), ALT, AST, BUN, C-reactive protein (CRP), White blood cell count (WBC)
Yi et al.[29]	2024	prospective study	26.80%	T2DM	1 month	Single-center	History of hypoglycemia, Insulin, Mini-Mental State Examination score (MMSE), HbA1c, education level, and duration of diabetes
Yang et al.[30]	2022	retrospective study	9.4%	T2DM	–	Single-center	Insulin, Sex, Respiratory rate (RR), Procalcitonin (PCT), and Hemoglobin (Hb)
Gong et al.[31]	2024	retrospective study	14.3%	T2DM	6 hours	Single-center	Continuous glucose monitoring (CGM) time-series
Shi et al.[32]	2024	retrospective study	4%	T2DM T1DM	12 months	Multicenter	Non-use of lipid-regulating drugs, in-patient admission, urgent emergency triage, Insulin, and history of hypoglycemia
Shao et al.[33]	2024	retrospective study	18.8%	T2DM T1DM	30min	Multicenter	CGM time-series
Liu et al.[34]	2026	retrospective study	25.5%	T2DM	6 hours	Single-center	Sex, drinking, BMI, Insulin, Age, and CGM
Liu et al.[35]	2025	retrospective study	18.85%	T2DM	–	Single-center	CP, insulin, BMI, LOS, Age, renal dysfunction, Albumin (ALB), lipohypertrophy, and insulin antibodies
Li et al.[36]	2025	retrospective study	14.69	T2DM T1DM	–	Single-center	BMI, Insulin, HbA1c, Meal omission, Cr, BUN, TG, ALB, ALT, Indirect bilirubin (IDIL), and Urine protein (PRO)
Jia et al.[37]	2025	retrospective study	41.82	T2DM	–	Single-center	Age, Charlson comorbidity index (CCI), Cr, HbA1c, and Insulin

### 3.3 Model development and validation

All included studies developed ML-based models to predict hypoglycemia. The sample sizes of the modeling datasets ranged from 192 to 255,404 participants, with validation datasets ranging from 70 to 109,459 participants. A variety of modeling approaches were applied, including logistic regression (LR), random forest (RF), support vector machine (SVM), decision tree (DT), extreme gradient boosting (XGBoost), Light Gradient Boosting Machine (LightGBM), deep neural networks (DNN), and long short-term memory (LSTM) networks. Ensemble learning algorithms, particularly XGBoost and RF, were frequently reported as the optimal models, with AUC values ranging from 0.822 to 0.978. Seven studies conducted external validation, whereas the remaining studies performed internal validation using k-fold cross-validation or bootstrapping. Regarding model performance evaluation beyond discrimination, six studies reported calibration assessment using calibration curves (n = 5) or the Hosmer–Lemeshow test (n = 1). However, none of the included studies reported decision-curve analysis or other measures of clinical utility. Detailed information on model characteristics, predictors, and validation is provided in Table 2.

**Table 2:** Model construction method and predictive performance.

Study	Modeling dataset	Validation dataset	Modeling methods	Validation method	Internal validation methods	Model performance	
						AUC	Calibration
Zhang et al.[25]	300	90	RF*	External verification	–	0.823 (0.752, 0.894)	Calibration curve
Zheng et al.[26]	428	184	Xgboost*, LR, RF, DT	Internal verification	K-fold cross-validation	0.910(0.845, 0.953)	–
Niu et al.[27]	2135	300	SVM, LR, RF*	External verification	–	0.931(0.911, 0.959)	–
Zuo et al [28]	840	360	LR, SVM, DT, RF, GBDT	Internal verification	K-fold cross-validation	0.880(0.821, 0.939)	–
Yi et al.[29]	233	70	LR*, DT	Internal verification	bootstrapping	0.860(0.808, 0.913)	Calibration curve
Yang et al.[30]	29,843	138	XGBoost*	External verification	–	0.822(0.786, 0.886)	Calibration curve
Gong et al.[31]	308	132	LR, RF, LightGBM*	External verification	bootstrapping	0.869(0.834, 0.891)	Hosmer-Lemeshow test
Shi et al.[32]	255,404	109,459	RF, GBM, DNN, XGBoost*	External verification	–	0.978(0.972, 0.984)	–
Shao et al.[33]	192	427	LSTM*, SVM, RF	External verification	–	0.972(0.968, 0.977)	–
Liu et al.[34]	1128	100	RF, ET, and KNN	External verification	–	0.926(0.0.913, 0.947)	Calibration curve
Liu et al.[35]	817	350	Xgboost*, LR, RF	Internal verification	K-fold cross-validation	0.853(0.834, 0.877)	–
Li et al.[36]	2,276	569	Xgboost, RF, DT, SVM, GBDT, AdaBoost, LightGBM, CatBoost*	Internal verification	–	0.850(0.814, 0.872)	Calibration curve
Jia et al.[37]	1,258	540	Xgboost, RF, LR	Internal verification	K-fold cross-validation	0.959(0.914, 0.972)	–

Note: DT = Decision Tree; DNN = Deep neural network; ET = Extra Trees; GBM = gradient boosting machine; GBDT = gradient boosting decision tree; KNN = K-Nearest Neighbors; LightGBM = Light Gradient Boosting Machine; LR = Logistic Regression; RF = Random Forest; SVM = Support Vector Machine; Xgboost = Extreme Gradient Boosting; * = optimal model.

### 3.4 Prevalence of hypoglycemia

**Fig 2:**
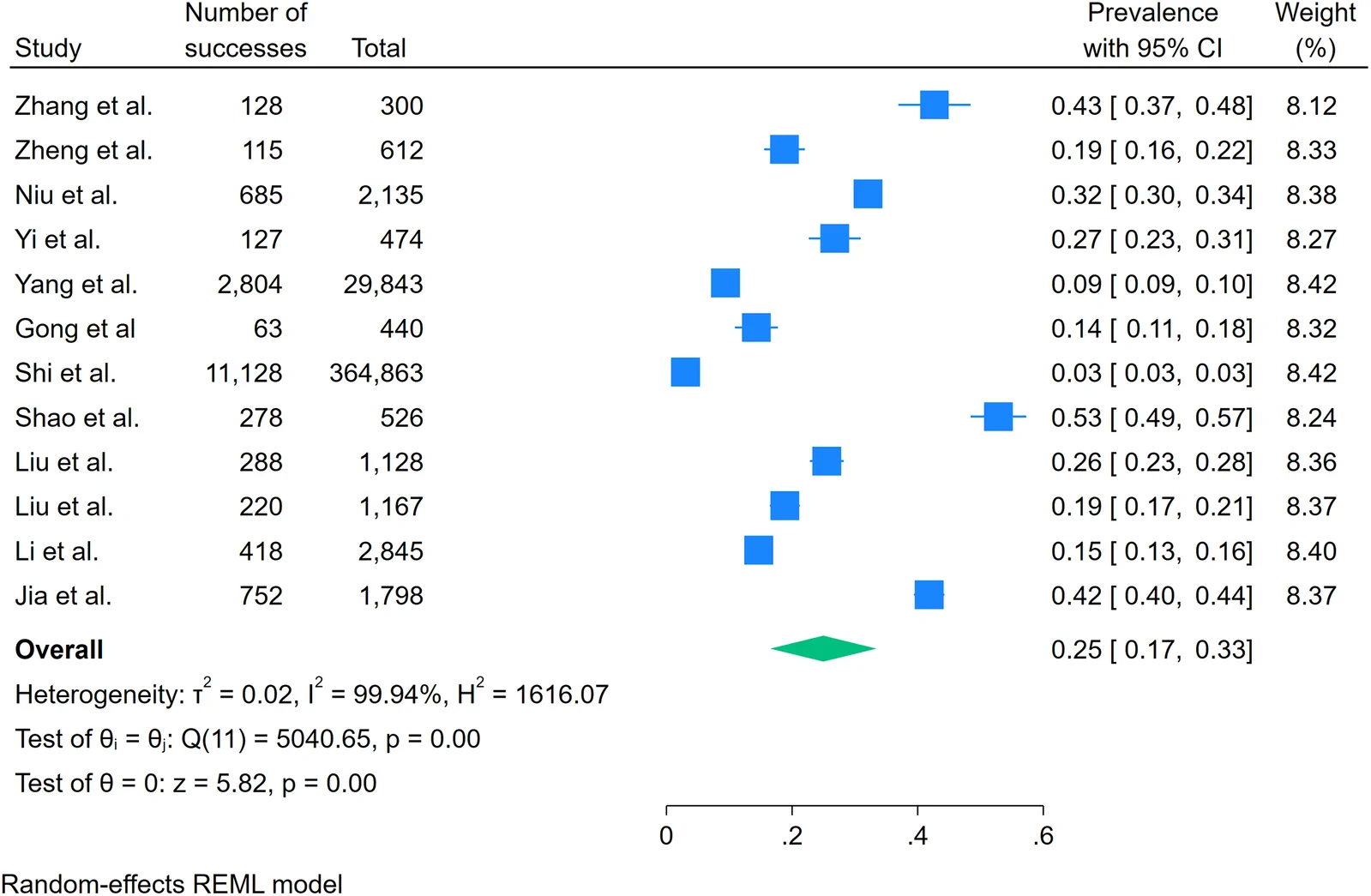
Forest plots for the pooled prevalence of hypoglycemia.

Among the 13 included studies, 12 reported the prevalence of hypoglycemia and were included in the meta-analysis. Using a random-effects model (REML), the pooled prevalence of hypoglycemia was estimated to be 25% (95% CI: 17%–33%). The detailed results are presented in the forest plot (Fig 2).

### 3.5 Predictors of the included models

Among the 13 included studies, all reported the predictors used in their hypoglycemia prediction models. Across the studies, more than 40 unique predictors were extracted. To highlight the most relevant variables, Table 3 presents only those predictors that were reported in at least two studies. The most frequently included predictors were age, insulin use, Meal omission, BMI, HbA1c, creatinine, and history of hypoglycemia, while the distribution of other predictors across the included models is summarized in the Table 3.

**Table 3:** Predictors in ML-based hypoglycemia models.

Predictor/ Study	Zhang 2023	Zheng 2023	Niu 2024	Zuo 2024	Yi 2022	Yang 2024	Gong 2023	Shi 2024	Shao 2024	Liu 2026	Liu 2025	Li 2025	Jia 2025
Age	★			★						★	★		★
Sex	★					★				★			
BMI			★	★						★	★	★	
Duration of diabetes	★		★		★								
History of hypoglycemia	★		★		★			★					
Insulin use	★		★		★	★		★		★	★	★	★
HbA1c	★				★							★	★
Cr		★		★								★	★
BUN		★		★								★	
TG			★	★								★	
HDL-C	★	★		★									
ALT			★	★								★	
AST			★	★									
ALB											★	★	
LOS			★								★		
C-peptide		★									★		
Meal omission			★									★	
CGM time-series							★		★	★			

### 3.6 Results of meta-analysis

#### 3.6.1 Meta-analysis of performance measures.

**Fig 3:**
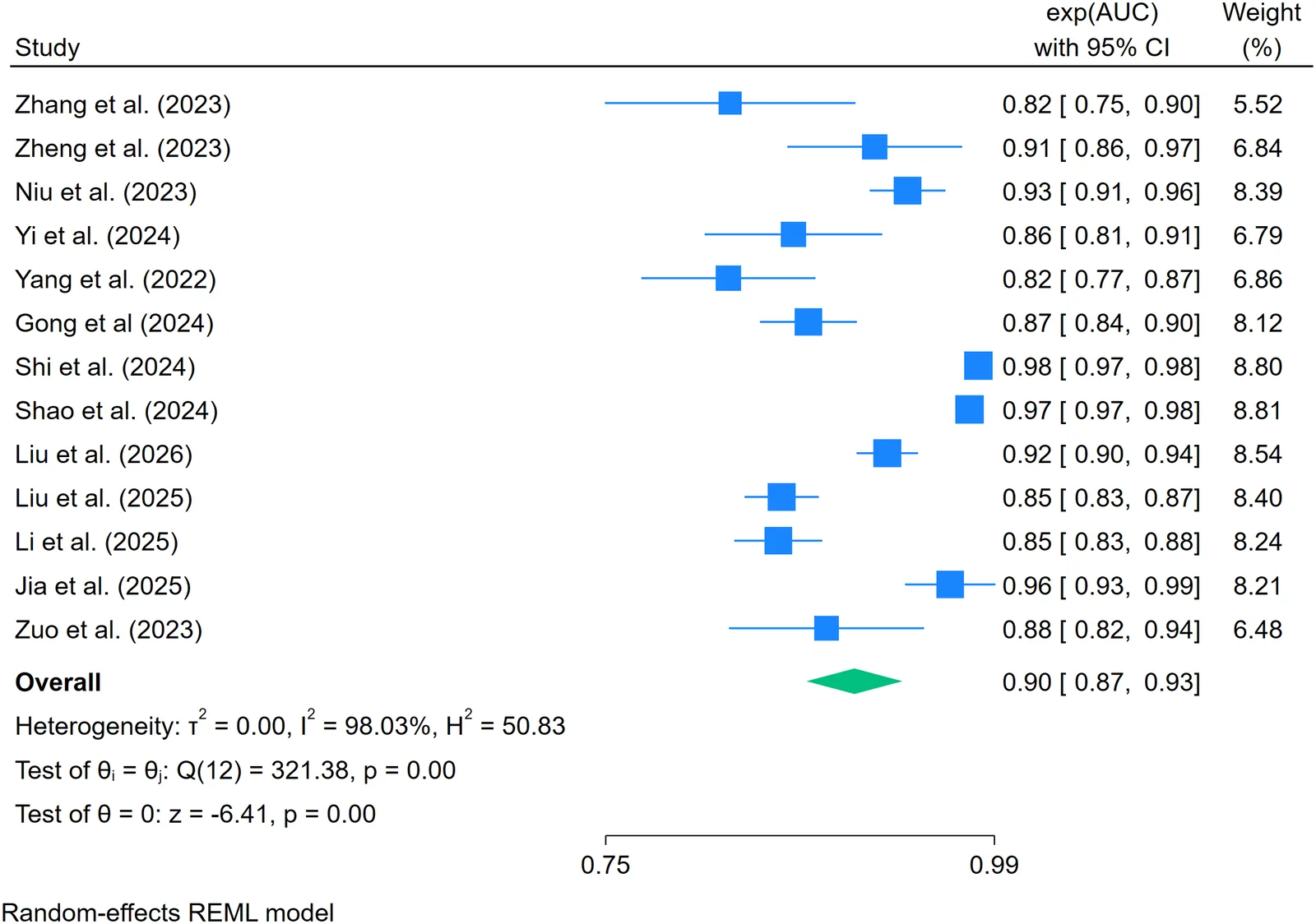
Forest plot of the AUC for the risk prediction model.

**Fig 4:**
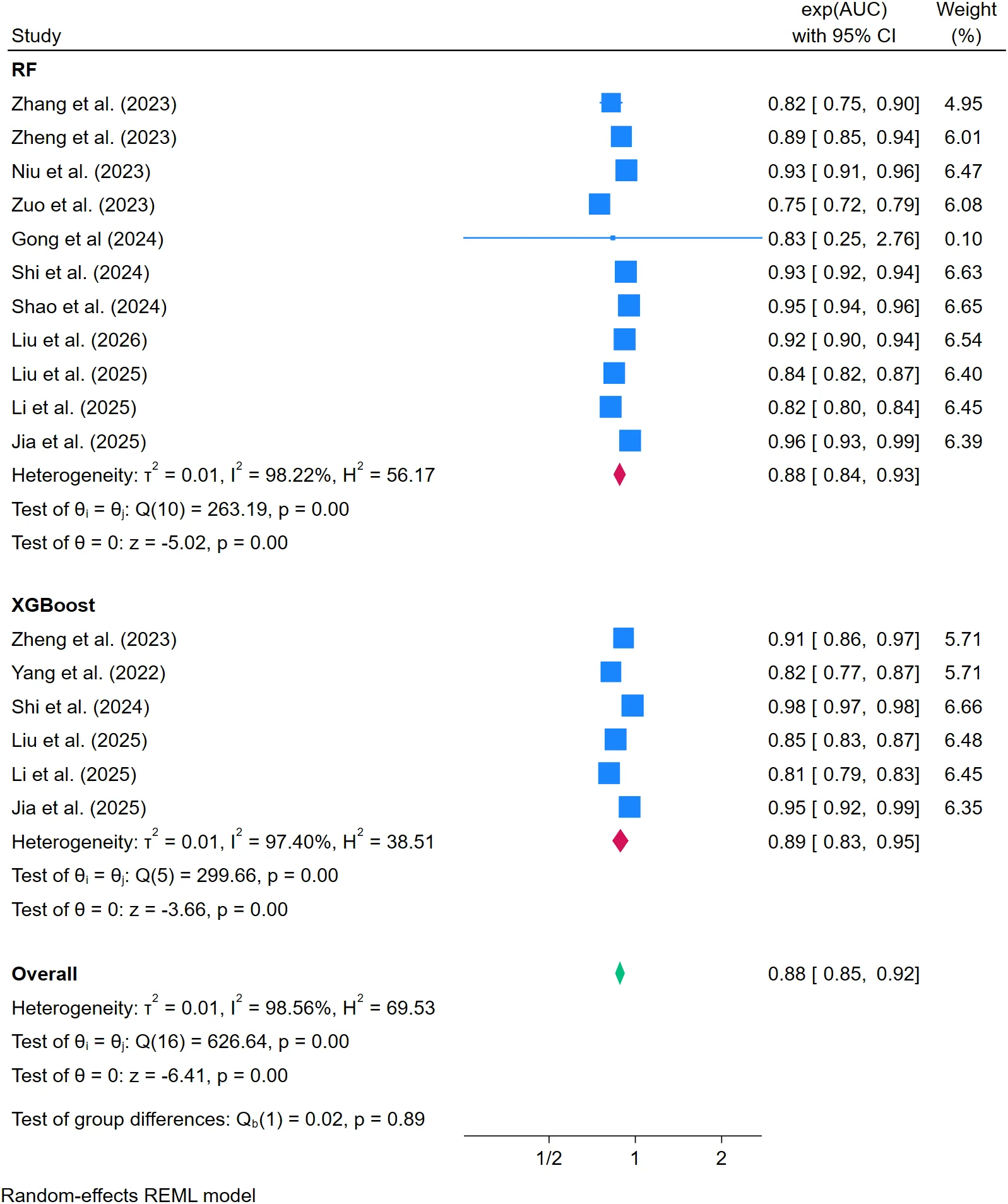
Forest Plot of Pooled AUCs for RF and XGBoost.

**Fig 5:**
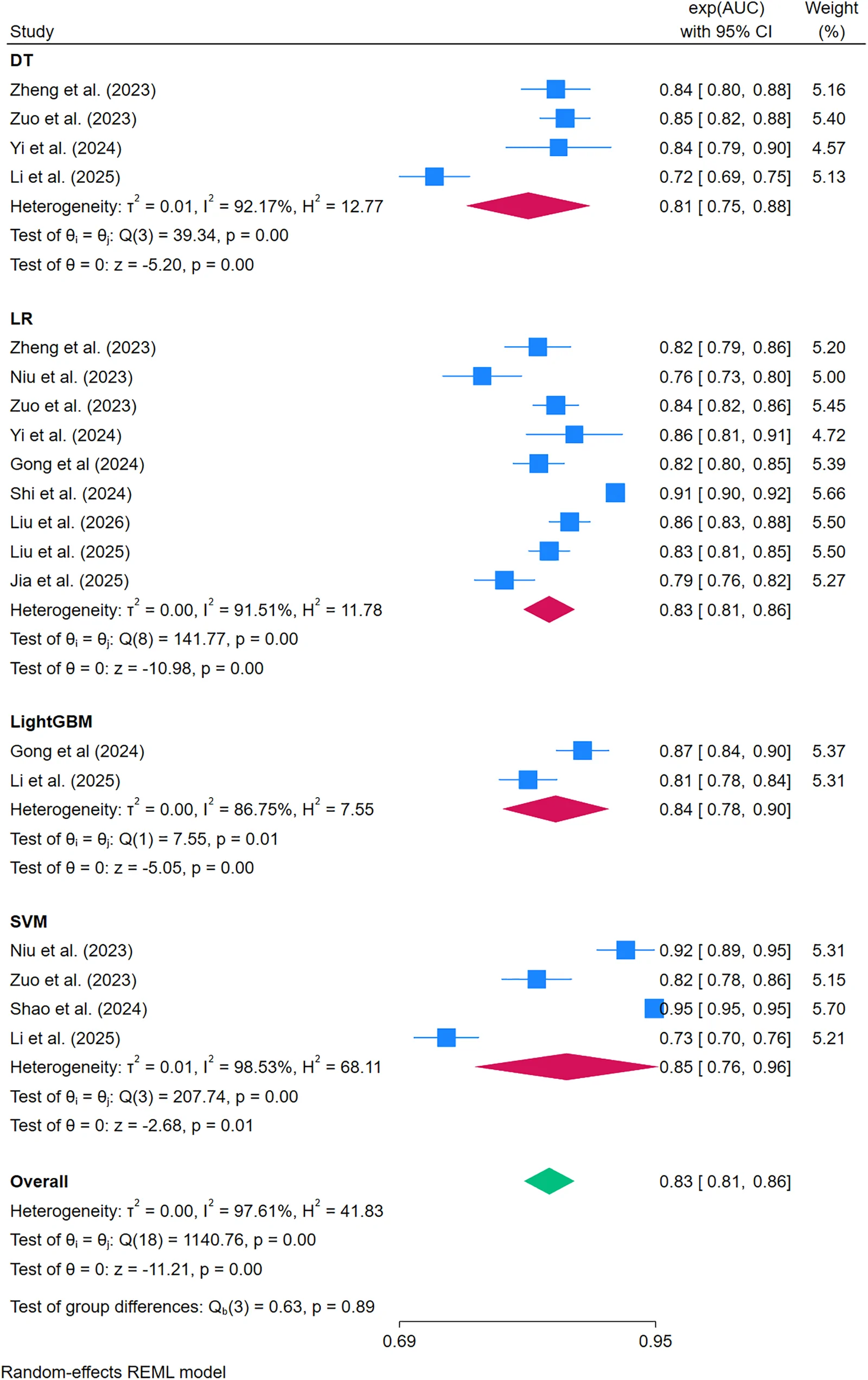
Forest Plot of Pooled AUCs for DT, LR, LightGBM, and SVM Models.

The forest plot summarizes the AUC values of the included studies. The pooled analysis yielded an overall AUC of 0.90 (95% CI: 0.87–0.93) (Figs 3 and 4). Subgroup analyses were further conducted according to modeling algorithms. The pooled AUCs were 0.84 (95% CI: 0.78–0.90; I^2^ = 86.75%, p < 0.001) for LightGBM, 0.89 (95% CI: 0.83–0.95; I^2^ = 97.40%, p < 0.001) for XGBoost, 0.88 (95% CI: 0.84–0.93; I^2^ = 98.22%, p < 0.001) for RF, 0.81 (95% CI: 0.75–0.88; I^2^ = 92.17%, p < 0.001) for DT, 0.83 (95% CI: 0.81–0.86; I^2^ = 91.51%, p < 0.001) for LR, and 0.85 (95% CI: 0.76–0.96; I^2^ = 98.53%, p < 0.001) for SVM (Figs 4 and 5).

#### 3.6.2 Subgroup of meta-analysis.

The subgroup analyses were done in accordance to study design, center type, predictor type and validation method (Table 2). When stratified by study design, the pooled AUC was 0.88 (95% CI: 0.86–0.91; I^2^ = 96.07%) for retrospective studies and 0.85 (95% CI: 0.80–0.89; I^2^ = 0.11%) for prospective studies. In the analysis by center type, the pooled AUC was 0.88 (95% CI: 0.85–0.91; I^2^ = 86.03%) for single-center studies and 0.87 (95% CI: 0.87–0.88; I^2^ = 59.42%) for multicenter studies. When stratified by predictor type, models developed using clinical variables showed a pooled AUC of 0.88 (95% CI: 0.85–0.90; I^2^ = 88.93%), whereas models based on CGM time-series data yielded a pooled AUC of 0.89 (95% CI: 0.85–0.94; I^2^ = 95.50%). According to validation method, models with internal validation demonstrated a pooled AUC of 0.90 (95% CI: 0.86–0.93; I^2^ = 78.53%), while those with external validation showed a pooled AUC of 0.87 (95% CI: 0.85–0.89; I^2^ = 95.70%) (Table 4).

**Table 4:** Subgroup Analyses of Pooled AUCs for Hypoglycemia Prediction Models.

Subgroup	No. of studies	AUC (95% CI)	P value	I² (%)	Effect model
Study design					
Retrospective study	11	0.88 (0.86–0.91)	<0.001	96.07	Random-effects
Prospective study	2	0.85 (0.81–0.89)	0.420	0.110	Fixed-effect
Center type					
Single-center	11	0.88 (0.85–0.91)	<0.001	86.03	Random-effects
Multicenter	2	0.87 (0.87–0.88)	0.12	59.42	Fixed-effect
Predictor type					
Clinical variables	11	0.88 (0.85–0.90)	<0.001	88.93	Random-effects
CGM time-series	2	0.89 (0.85–0.94)	<0.001	95.50	Random-effects
Validation method					
Internal verification	6	0.90 (0.86–0.93)	<0.001	78.53	Random-effects
External verification	7	0.87 (0.85–0.89)	<0.001	95.70	Random-effects

### 3.7 Quality assessment of included studies

A PROBAST tool was used to evaluate the risk of bias and applicability of the included studies (Table 5). Out of 13 included studies, 4 studies were found to be at low overall risk of bias, 2 were found to be at unclear risk of bias, and the rest of the studies were found to be at high risk of bias. In the domain level assessment, most studies had low risk of bias in participants and predictors domains, but some studies had issues in outcome and analyses domains. Concerning applicability, most studies presented low concerns in participants and predictors domains, and a small number of studies had high or unclear concerns in outcome domain.

**Table 5:** Risk of bias assessment.

Study	Risk of bias				Applicability			Overall	
	Participants	Predictors	Outcome	Analysis	Participants	Predictors	Outcome	ROB	Applicability
Zhang et al.	+	+	+	+	+	+	+	+	+
Zheng et al.	+	+	?	−	+	+	?	−	−
Niu et al.	+	+	+	+	+	+	?	+	+
Zuo et al.	+	+	?	+	+	+	?	?	+
Yi et al.	+	+	?	+	+	+	?	?	+
Yang et al.	+	+	+	+	+	+	+	+	+
Gong et al	+	+	−	+	+	+	+	−	+
Shi et al.	+	+	+	+	+	+	+	+	+
Shao et al.	+	+	?	−	+	+	−	−	+
Liu et al.	+	+	?	−	+	+	−	−	+
Liu et al.	+	−	?	−	+	+	+	−	+
Li et al.	+	−	−	−	+	−	−	−	?
Jia et al.	+	+	−	−	+	+	−	−	+

ROB = risk of bias. “+” indicates low risk of bias or low concern regarding applicability; “−” indicates high risk of bias or high concern regarding applicability; “?” indicates unclear risk of bias or unclear concern regarding applicability.

### 3.8 Sensitivity analysis and publication bias

**Fig 6:**
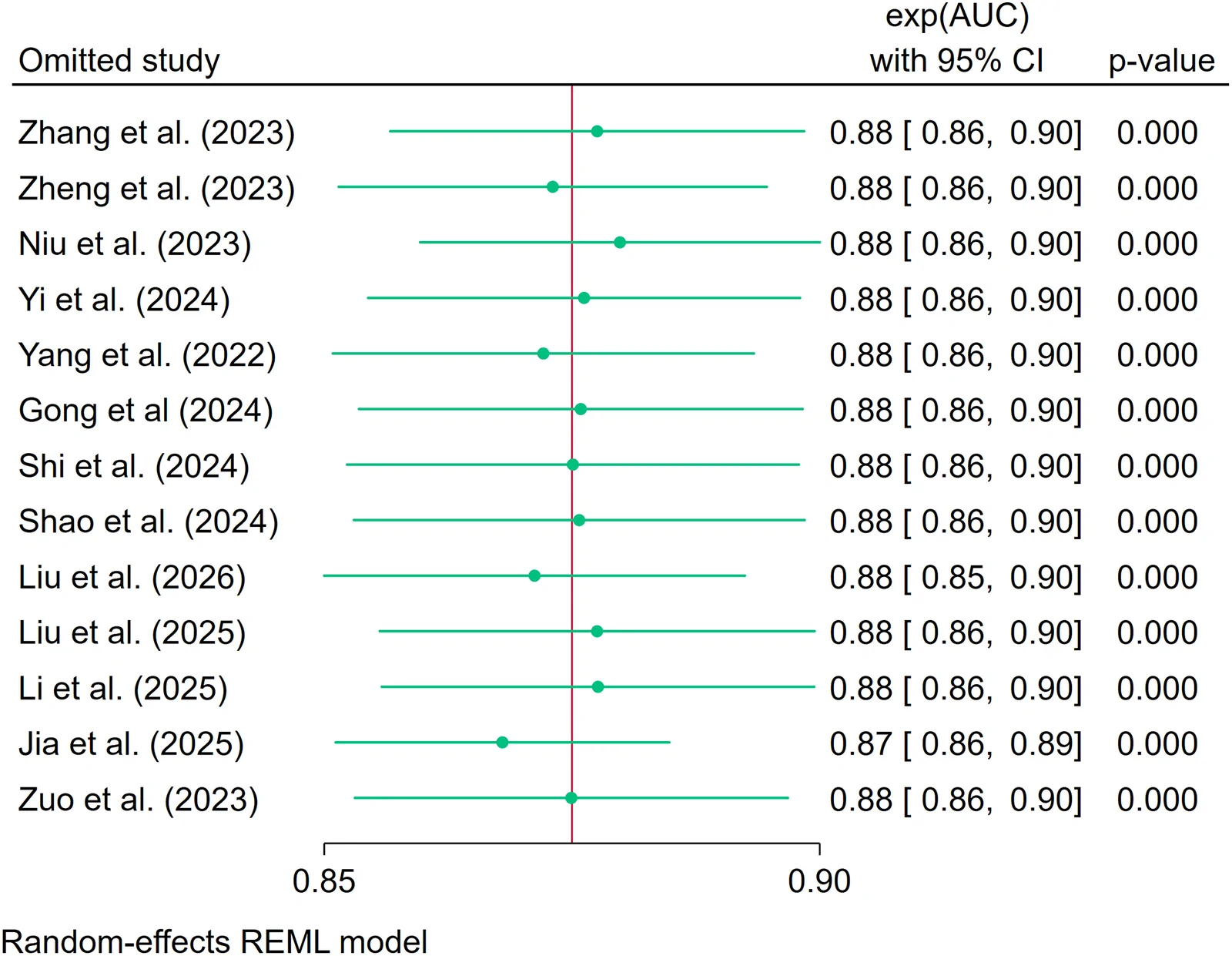
Sensitivity Analysis.

**Fig 7:**
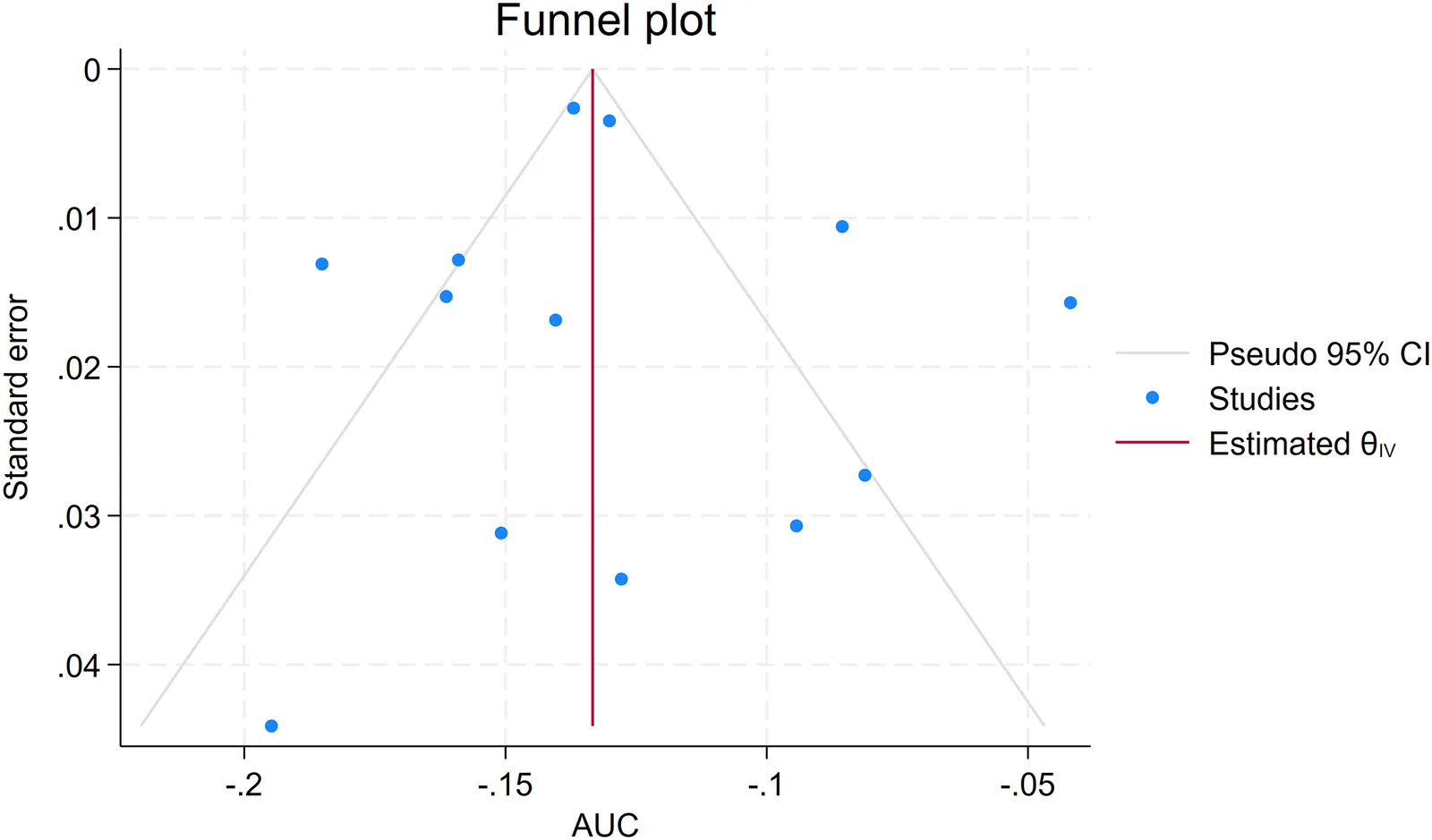
Funnel plot examination.

The sensitivity analysis was performed by the leave-one-out approach and the pooled findings did not significantly change when each study was sequentially excluded which means that there is no one study that has played a significant role in the overall estimate (Fig 6). Visual inspection of the funnel plot and Begg test were used to evaluate publication bias. Begg test gave a P value of 0.669, which indicates no statistically significant evidence of publication bias. Most of the studies were found to be located at the expected funnel area, and the funnel plot was almost symmetrical, indicating no obvious presence of publication bias in this meta-analysis (Fig 7).

## 4 Discussion

### 4.1 Predictive performance and methodology

With the rapid aging of the population and changes in dietary patterns, the prevalence of diabetes in China has increased substantially in recent years [38], leading to a growing burden of hypoglycemia and its associated adverse outcomes [3]. In the present meta-analysis, the pooled prevalence of hypoglycemia among Chinese patients with diabetes was 25% (95% CI: 17%–33%), highlighting the clinical burden of hypoglycemia in this population. China has the largest population of individuals with diabetes worldwide, and most affected patients have type 2 diabetes mellitus (T2DM). Given the large population size and specific clinical and healthcare contexts of Chinese patients with diabetes, prediction models developed or validated in this population may provide more relevant evidence for hypoglycemia risk assessment and clinical application. In parallel, advances in artificial intelligence have facilitated the development of numerous prediction models aimed at identifying patients at high risk of hypoglycemia [39]. To our knowledge, this study represents the first systematic review focusing on hypoglycemia prediction models specifically in Chinese patients with diabetes. Notably, the studies included were all published within the past five years, reflecting the most recent developments in this field. Most of the included studies focused on patients with type 2 diabetes; although some studies included a small number of patients with type 1 diabetes, their sample sizes were limited, and the results primarily reflect hypoglycemia risk in type 2 diabetes. In addition, we applied the PROBAST tool to comprehensively assess the risk of bias and conducted publication bias evaluation, which together support the overall methodological rigor and reliability of the present study.

The performance of the models in the included studies was mainly assessed through the AUC, the most commonly used measure of discrimination in prediction model studies. Combined results indicated a pooled AUC of 0.90 (95 percent confidence interval: 0.87–0.93), and this implied that overall predictive efficacy of available hypoglycemia risk models was satisfactory to good. However, AUC only reflects the discrimination ability of prediction models and cannot fully represent their calibration or clinical usefulness. In this meta-analysis, only a limited number of studies reported calibration assessment, and none evaluated clinical utility using decision-curve analysis, indicating that further studies are needed to comprehensively evaluate the reliability and clinical applicability of these models.Analysis of subgroups, according to modeling methods also showed variation in predictive performance between different model types. Machine learning-based models (XGBoost and RF) were found to be comparably more accurate in their predictions than the conventional logistic regression models. This finding is consistent with previous studies [16,40], which has indicated that ML methods are likely to outperform traditional statistical models due to their ability to identify more complicated nonlinear relationships and relationships between predictors.

Nevertheless, despite the overall beneficial discrimination, there was significant heterogeneity among the included studies. It is typical in meta-analyses of prediction models and can be explained by differences in study designs, patient populations, choice of predictors, outcomes, and modeling approaches [41]. In order to investigate possible causes of heterogeneity, subgroup analyses were performed according to study design, type of center, and validation method. It was found that externally validated models tended to give lower AUC values than internally validated models. This inconsistency can be justified by the statement that internal validation usually generates optimistic estimates of model performance and external validation, which is carried out in independent datasets, gives a more accurate estimate of the model generalizability and robustness [41]. The remaining subgroup analyses have not shown significant discrepancies in predictive performance. This could be in part because the number of studies in some subgroups was low, most of the studies included in this analysis were retrospective and single-center, but there were fewer prospective and multi-center studies. These disparities in the characteristics of studies might hinder the possibility to recognize the actual differences between subgroups. Additionally, using retrospective data can lead to selection bias and reduce the representativeness of the study populations. Due to the diversity of clinical practice, patient features, and healthcare facilities, it is important to exercise caution when interpreting the pooled findings and extrapolating these models into more general clinical settings.

### 4.2 Predictors of hypoglycemia risk in prediction models

Even though there was a variation in terms of modeling approaches, sources of data and population of studies, a significant level of uniformity was noted in the choice of predictors. The total number of candidate predictors identified in the 13 included studies was over 30, where age, insulin use, BMI, HbA1c, creatinine, and hypoglycemia history were the most prevalent variables. Such a pattern indicates the development of a rather steady list of core predictors of hypoglycemia risk evaluation. For example, Zhang et al. [25] stated age as a significant predictor and indicated that elderly individuals were significantly predisposed to hypoglycemia, which can be explained by the lack of adequate counter-regulatory response, other comorbidities, and decreased resistance to glucose-lowering treatment. Similarly, Yang et al. [30] also considered the age in their model and showed its great contribution, further strengthening its constant importance in risk prediction. Another significant predictor that was continuously used in the models was the use of insulin. Li et al. [36] reported that patients receiving insulin therapy had a markedly increased risk of hypoglycemia, which is consistent with the pharmacological effect of insulin and the complexity of dose adjustment. Likewise, Liu et al. [35], using machine learning methods, identified insulin use as a major contributor to model performance, indicating its robust predictive value across different modeling strategies. In addition, a history of hypoglycemia is widely recognized as one of the strongest predictors of future events. Zuo et al. [28] demonstrated that prior hypoglycemic episodes were associated with a substantially increased risk of recurrence, possibly due to impaired hypoglycemia awareness and individual susceptibility. This finding was further supported by Yi et al. [29], who also incorporated this variable into their model and confirmed its importance in prediction. Regarding metabolic and laboratory indicators, BMI, HbA1c, and creatinine were also frequently reported. Zheng et al. [26] found that lower BMI was associated with a higher risk of hypoglycemia, which may reflect reduced energy reserves and diminished glycogen storage. Niu et al. [27] similarly identified BMI as a contributing factor in their model, suggesting a role for nutritional status in hypoglycemia risk. HbA1c, a marker of long-term glycemic control, was consistently included across multiple studies. Gong et al. [31] reported that lower HbA1c levels were associated with an increased risk of hypoglycemia, likely reflecting the effects of intensive glycemic management and greater glucose variability. This was further corroborated by Shi et al. [32], who demonstrated that HbA1c contributed meaningfully to model discrimination. Creatinine, as an indicator of renal function, was also incorporated in several models. Shao et al. [33] reported that impaired renal function was associated with an elevated risk of hypoglycemia, potentially due to reduced clearance of insulin and oral hypoglycemic agents, thereby prolonging their effects. Jia et al. [37] likewise included creatinine in their model and found it to contribute to predictive performance, underscoring the relevance of renal function in hypoglycemia risk assessment. However, some clinically important factors were not consistently included in the prediction models. For example, sulfonylurea use and excessive doses of glucose-lowering medications, which are known risk factors for hypoglycemia, were rarely reported among the included studies. This may be related to differences in data availability, medication information collection, and variable selection methods across studies. Future studies should consider incorporating more clinically relevant factors to improve the prediction models.

Beyond these commonly reported predictors, CGM have gained increasing attention in recent years and represent a distinct category compared with traditional clinical variables [42]. Unlike static demographic or laboratory indicators, CGM provides high-resolution, time-series data that capture dynamic glucose fluctuations, offering valuable insights into short-term glycemic trends preceding hypoglycemic events [43]. With advances in artificial intelligence, an increasing number of studies have leveraged CGM time-series data in combination with machine learning or deep learning algorithms to develop hypoglycemia prediction models [44–46]. Some studies have reported that such models achieve improved predictive accuracy and are capable of forecasting hypoglycemic events within short time horizons (e.g., up to 30 minutes in advance), thereby enabling timely clinical intervention. However, in the present meta-analysis, no significant difference in AUC was observed between models based on CGM data and those using conventional clinical variables. This finding should be interpreted with caution, as only two studies incorporating CGM data were included, limiting both representativeness and statistical power. As a result, the potential advantage of CGM-based models may not have been fully captured. Therefore, the comparative impact of CGM-derived versus traditional clinical predictors on model performance remains uncertain. Further high-quality studies, particularly those based on large, multicenter datasets, are needed to clarify the added value of CGM and its derived features in improving hypoglycemia prediction.

### 4.3 Implications for future research

Notable progress has been made in the validation of hypoglycemia prediction models, particularly in external validation. Seven included studies performed external validation, indicating increasing attention to model transportability across clinical settings and supporting their translation into clinical practice. Future research should further standardize methodological processes in model development and evaluation. Internal validation strategies need to be clearly reported, including detailed descriptions of methods such as cross-validation and bootstrapping, to improve transparency in assessing model stability. In addition to discrimination metrics, model performance should be evaluated more comprehensively by incorporating measures that assess the agreement between predicted and observed outcomes, thereby providing stronger support for reliable clinical application. At the same time, greater emphasis should be placed on model interpretability and presentation. Most included models were reported as regression coefficients or combinations of predictors, with limited use of intuitive tools such as nomograms or web-based calculators, and inadequate characterization of feature contributions. Incorporating explainable approaches, such as Shapley Additive Explanations (SHAP), along with user-friendly tools like nomograms, would enable quantification of predictor contributions and their directional effects, thereby enhancing model interpretability [47]. Presenting models through user-friendly formats, including nomograms, interactive web tools, or embedded systems, may further facilitate individualized risk assessment and improve usability in clinical practice. Furthermore, advances in health information technology provide opportunities to integrate prediction models into clinical workflows. Embedding these models into electronic health records or glucose management platforms could enable automated risk calculation and real-time alerts, reducing clinician workload and improving efficiency. Beyond risk estimation, the clinical value of prediction models should extend to guiding intervention strategies. In line with current diabetes management guidelines that emphasize risk stratification for hypoglycemia prevention, future research should explore how model outputs can inform stratified management approaches, such as optimizing glucose-lowering regimens, adjusting insulin dosing, or intensifying glucose monitoring in high-risk patients [48]. This would support the transition from risk prediction to risk-informed clinical decision-making.

Finally, future research should prioritize external validation of existing models rather than developing new ones. Models may be locally validated according to regional population characteristics and healthcare settings, and updated using multicenter data to improve real-world performance. These efforts would facilitate the clinical use of hypoglycemia prediction models and support early identification and management.

### 4.4 Limitations

This study has several limitations. Although a systematic and comprehensive search strategy was used, the literature search was restricted to articles published in English and Chinese, which may have resulted in the omission of relevant studies published in other languages. The included studies varied in study design, sample size, predictors, and modeling methods, which may have contributed to the substantial heterogeneity observed in the meta-analysis. In addition, although CGM provides valuable information for hypoglycemia prediction, false low glucose readings caused by compression during sleep may occur and affect the identification of hypoglycemia. The majority of the models were developed based on retrospective data and were mainly internally validated, with limited external validation, which may restrict their generalizability across different clinical settings and populations. Furthermore, this review focused on model characteristics and predictive performance, while clinical utility was not fully assessed due to variations in clinical contexts and implementation conditions. Therefore, the pooled findings should be interpreted cautiously, and further prospective studies with large-scale multicenter external validation are needed to confirm the clinical applicability of these models.

## 5 Conclusion

This study aimed to identify the existing literature on prediction models for hypoglycemia in Chinese with diabetes. A total of 13 models were included, showing considerable variation in predictors, modeling approaches, validation strategies, and performance reporting. The current evidence suggests that most models achieve acceptable discriminatory ability, and some have undergone external validation, indicating gradual progress toward potential clinical application. The scientific literature on ML–based prediction of hypoglycemia is steadily developing, and there is a great potential for these models to support early risk identification and clinical decision-making, but factors such as study design, validation strategies, calibration reporting, and model transparency need to be carefully considered.

## Supporting information

S1 Checklist PRISMA_2020_checklist. (DOCX)

S1 File Data. (XLSX)
